# The significance of wrist immobilization for endoscopic carpal tunnel release

**DOI:** 10.3389/fneur.2023.1081440

**Published:** 2023-04-25

**Authors:** Fei Zhang, Hong Jiang, Zhenfeng Lu, Haoyu Yang, Qian Zhang, Jingyi Mi, Yongjun Rui, Gang Zhao

**Affiliations:** ^1^Department of Hand Surgery, WuXi 9th People's Hospital Affliated to SooChow University, Wuxi, China; ^2^Suzhou Medical College of Soochow University, Soochow University, Suzhou, China; ^3^Department of Sport Medicine, WuXi 9th People's Hospital Affliated to Soochow University, Wuxi, China; ^4^Department of Orthopeadics Surgery, WuXi 9th People's Hospital Affliated to Soochow University, Wuxi, China

**Keywords:** carpal tunnel syndrome, median nerve, transverse carpal ligament, endoscopic carpal tunnel release, immobilization

## Abstract

**Background:**

Over the years, endoscopic carpal tunnel release (ECTR) has gained significant interest as an alternative to surgery. However, no consensus has been reached on the necessity of postoperative wrist immobilization. This study aims to compare the outcomes of wrist immobilization for a period of 2 weeks to immediate wrist mobilization after ECTR.

**Methods:**

A total of 24 patients with idiopathic carpal tunnel syndrome undergoing dual-portal ECTR from May 2020 to Feb 2022 were enrolled and randomly divided into two groups postoperatively. In one group, patients wore a wrist splint for 2 weeks. In another group, wrist mobilization was allowed immediately after surgery. The two-point discrimination test (2PD test); the Semmes–Weinstein monofilament test (SWM test); the occurrence of pillar pain, digital and wrist range of motion (ROM); grip and pinch strength; the visual analog score (VAS), the Boston Carpal Tunnel Questionnaire (BCTQ) score; the Disabilities of the Arm, Shoulder, and Hand (DASH) score; and complications were evaluated at 2 weeks and 1, 2, 3, and 6 months after the surgery.

**Results:**

All 24 subjects finished this study with no dropouts. During the early follow-up, patients with wrist immobilization demonstrated lower VAS scores, lower occurrence of pillar pain, and higher grip and pinch strength compared with the immediate mobilization group. No significant difference was obtained between these two groups in terms of the 2PD test, the SWM test, digital and wrist ROM, BCTQ, and the DASH score. In total, two patients without splints reported transient scar discomfort. No one complained of neurapraxia, injury of the flexor tendon, median nerve, and major artery. At the final follow-up, no significant difference was found in any parameters between both groups. The local scar discomfort mentioned above disappeared and left no serious sequela.

**Conclusion:**

Wrist immobilization during the early postoperative period demonstrated significant pain alleviation along with stronger grip and pinch strength. However, wrist immobilization yielded no obvious superiority regarding clinical outcomes at the final follow-up.

## Introduction

Carpal tunnel syndrome (CTS) is the most common peripheral compressive neuropathy worldwide (1). Carpal tunnel release (CTR) represents an alternative approach indicated when conventional intervention fails or symptoms recur (2). The surgery can be undergone via the traditional open or endoscopic approach (3–5). Although endoscopic carpal tunnel release (ECTR) yields similar clinical outcomes compared to open release, ECTR has gained significant attention worldwide for its improved aesthetics and fast recovery (5–9).

However, postoperative immobilization is still subject to much controversy. Earlier studies showed comparable outcomes and even worse prognosis of splint immobilization after open release compared with initial active motion (10–19). According to the 2016 American Academy of Orthopedic Surgeons (AAOS) evidence-based guidelines, postoperative immobilization yields no benefit (20). There are no valid data and associated criteria on splint immobilization after ECTR until now. This study sought to evaluate the outcomes of splint immobilization or initial active motion after ECTR and assess the significance of postoperative wrist immobilization.

## Materials and methods

This study was approved by the Ethics Committee of WuXi No.9 People's Hospital Affiliated to Soochow University. All subjects provided informed consent before this study started.

### Subjects

Outpatients at the WuXi No.9 People's Hospital Affiliated to Soochow University from May 2020 to Feb 2022 diagnosed with CTS clinically or by electrodiagnostic testing were recruited in our study. Inclusion criteria were severe idiopathic CTS with an electromyography (EMG) test and failure of conventional intervention or symptom recurrence. We defined severe CTS according to the AAEM grading criteria: prolonged median motor and sensory distal latencies, with either an absent SNAP or mixed NAP or low amplitude or absent thenar CMAP. Needle examination often reveals fibrillations, reduced recruitment, and motor unit potential changes (21). Exclusion criteria were slight or moderate CTS, mass-like cyst in the carpal tunnel, other wrist disorders, such as arthritis, wrist fracture within 3 months, CTS revision, and local skin or subcutaneous tissue disorders. Ultimately, 24 subjects according to the inclusion and exclusion criteria were enrolled and randomly divided into two groups, each containing 12 subjects. In total, five subjects had bilateral CTS and received ECTR concomitantly.

### Intervention

All subjects received one sealed envelope prepared by a person who was not involved in this trial after inclusion. The envelope contained the subject's sequential number and group and was opened just before the skin preparation of the surgical area. All subjects underwent dual-portal ECTR by the same operator. After surgery, one group was provided with a plaster splint put in the volar side of the wrist and half of the forearm and wrapped with bandage gauze. The wrist was immobilized in the neutral position for 2 weeks while the metacarpophalangeal joint and elbow could be moved immediately after surgery. Thereafter, the splint and bandage were removed after 2 weeks at the outpatient. For another group, an elastic bandage was applied to cover the incision, with no limitation of active wrist motion. All subjects were discharged on the first or second postoperative day with no analgesics and were required to visit the outpatient clinic five times at 2 weeks and 1, 2, 3, and 6 months.

### Outcome measure

Outcome parameters contained the 2PD test, SWM tests, the occurrence of pillar pain, digital and wrist range of motion, grip and pinch strength, VAS for pain, BCTQ, DASH score, and complications. All data were measured and recorded by an independent therapist with the Jamar^®^ Hand Evaluation kit.

Sensibility testing was measured by the 2PD and SWM tests. The 2PD test was used to evaluate the threshold capacity to distinguish two different pressure points applied on the fingertip via a particular discriminator. The SWM test was used to evaluate the touch threshold of the fingertip via five standardized filaments (size 2.83, size 3.61, size 4.31, size 4.56, and size 6.65; size 2.83 represents normal sensibility, and size 6.65 represents no protective sensibility). The tactile senses of the first to fourth digital fingertips were measured three times, and the median value was obtained.

Postoperative pain was assessed by VAS and the occurrence of pillar pain. VAS of the wrist was measured during active motion without any load with a 100 mm visual analog scale ranging from 0 to 10 (0: absence of pain and 10: worst pain).

Postoperative strength was measured by the grip and pinch strength. Grip strength was measured in a seated position with the shoulder and wrist in a neutral position, with the elbow in a 90-degree flexed position, using the Jamar dynamometer. Key pinch strength measured the pressure between the thumb pad and the middle phalanx of the index digit with a pinch gauge dynamometer. All tests were measured three times, and the best value was recorded to decrease bias.

Postoperative flexibility was assessed by digital and wrist ROM. Each digital ROM was measured with a finger goniometer, and the interphalangeal joint of thumb value was recorded for further comparison. Wrist ROM during flexion and extension was measured in a seated position with elbow flexion and the hand placed in a resting position using a wrist goniometer.

Postoperative self-evaluation was measured by the BCTQ and DASH questionnaire. The BCTQ consists of two scales: a symptom severity scale (BCTQ-S) and a functional status scale (BCTQ-F) (22, 23). The former section contained 11 items with a score between 1 and 5 (one means normal and five means serious symptom), whereas the latter section included eight items regarding physical function with a score between 1 and 5 (one means no difficulty and five means that subject cannot perform the activity). The average value of each section was recorded for further comparison. The DASH questionnaire contained 30 items with a score between 1 and 5 (one means no difficulty and five means inability). The score was calculated with the formula [(Sum of *n* responses)/*n* – 1] ^*^ 25 and recorded for further comparison (24).

### Statistical analysis

Data were analyzed using SPSS software, v.25 (IBM SPSS Statistics 25, USA). Digital and wrist ROM and grip and pinch strength were analyzed using a Student's *t*-test. Data were expressed in the form of mean ± standard deviation. The 2PD test, SWM test, VAS, BCTQ, and DASH score were analyzed with Wilcoxon's rank sum test. Non-parametric data were expressed as median values. The occurrence of pillar pain was analyzed with the Fisher exact test. Data were expressed in the form of occurrence. The level of significance was set at a *P*-value of < 0.05.

## Results

The outcome measures were assessed in all subjects, and none were lost during the follow-up period. The subjects exhibited a female predominance (*n* = 18, 75%) aged 26–82 years old. The duration of symptoms ranged from 1 to 120 months. In addition to gender, there was no significant difference between the two groups regarding demographic data (Table 1).

**Table 1:** Group characteristics.

**Characteristics**	**All patients (*n* = 24)**	**WI group (*n* = 12)**	**AM group (*n* = 12)**	***P*-value**
**Gender (** ***n*** **)**				
Male/female	6/18	2/10	4/8	<0.01^*^
**Age (years)**				
Mean ± SD	49.96 ± 11.67	51.00 ± 11.54	48.92 ± 12.22	0.71
**Affected hand (** ***n*** **)**				
Left/right/bilateral	7/12/5	4/5/3	3/7/2	0.76
**Dominant hand (** ***n*** **)**				
Left/right	1/23	0/12	1/11	0.16
**Length of symptom (months)**				
Mean ± SD	12.63 ± 24.28	9.83 ± 9.36	15.42 ± 33.58	0.295

WI, wrist immobilization group; AM, active motion group; SD, standard deviation; n, number. ^*^ denotes results with statistical significance.

The 2PD and SWM tests showed an apparent improvement compared with preoperative values although no significant difference was found between both groups at each time point. Moreover, the wrist immobilization group yielded better VAS and lower occurrence of pillar pain compared with the wrist mobilization motion group at 2 weeks postoperatively. No difference in pillar pain was observed at 1 month postoperatively. In addition, nearly 80% of subjects were pain-free at the final follow-up in both cohorts. Moreover, grip and pinch strength decreased in early follow-up and got restored to preoperative levels at 2 months postoperatively. At 3 months postoperatively and the final follow-up, strength was slightly stronger than preoperative values in both groups. When these two groups were compared, the wrist immobilization group showed stronger grip and pinch strength than the wrist mobilization group at 2 weeks postoperatively. Subsequently, the strength of both groups was comparable. Digital and wrist ROM showed no obvious difference between preoperative and final follow-up and in both cohorts at any time. For the BCTQ and DASH questionnaire, both groups showed decreased scores at 2 weeks postoperatively and returned to preoperative levels at 1 month. Although the DASH scores of wrist immobilization at 1 month were greater, the difference showed no statistical significance. In total, two subjects in the wrist mobilization group complained of scar discomfort in the distal wound during early follow-up, which disappeared at 1 month postoperatively. No severe complications, including wound dehiscence, lesion of the superficial palmar arch, or median nerve, occurred during the whole follow-up. All data are shown in Tables 2–5.

**Table 2:** Outcome measures of strength and flexibility.

	**Pre M ±S**	**2 weeks M ±S**	**1 month M ±S**	**2 months M ±S**	**3 months M ±S**	**6 months M ±S**
**D-ROM (**°**)**						
WI	96.73 ± 7.91	88.40 ± 6.36	93.73 ± 6.03	97.07 ± 7.64	98.00 ± 7.21	98.40 ± 6.38
AM	95.14 ± 5.80	91.00 ± 4.67	92.86 ± 4.79	95.57 ± 5.33	96.43 ± 5.17	96.64 ± 5.53
*P*-value	0.55	0.22	0.67	0.55	0.51	0.44
**W-ROM (**°**)**						
WI	146.60 ± 11.98	116.60 ± 13.51	138.80 ± 11.42	146.13 ± 12.94	146.53 ± 13.08	148.40 ± 11.28
AM	148.36 ± 6.95	117.57 ± 7.52	140.57 ± 5.83	148.64 ± 7.23	149.50 ± 6.75	150.57 ± 6.73
*P*-value	0.64	0.81	0.61	0.53	0.45	0.54
**Grip (kg)**						
WI	24.40 ± 7.29	17.40 ± 4.63	20.40 ± 4.72	26.67 ± 8.39	28.53 ± 7.84	29.27 ± 7.80
AM	27.93 ± 7.32	14.07 ± 2.95	18.79 ± 4.59	26.29 ± 7.25	28.36 ± 6.73	29.07 ± 6.47
*P*-value	0.21	0.03^*^	0.36	0.90	0.95	0.94
**Pinch (kg)**						
WI	6.83 ± 1.94	4.93 ± 1.28	5.63 ± 1.72	7.13 ± 1.90	7.80 ± 1.96	7.90 ± 2.06
AM	7.29 ± 2.40	3.71 ± 1.49	5.11 ± 2.44	7.29 ± 1.83	7.54 ± 1.88	7.64 ± 1.79
*P*-value	0.58	0.025^*^	0.51	0.83	0.71	0.72

Pre, preoperative; D-ROM, digital range of motion; W-ROM, wrist range of motion; WI, wrist immobilization group; AM, active motion group; M±S, mean ± standard deviation. ^*^ denotes results with statistical significance.

**Table 3:** Outcome measures of sensibility.

		**Pre median**	**2 weeks median**	**1 month median**	**2 months median**	**3 months median**	**6 months median**
2PD 1st digit	WI	4	4	3	3	3	3
	AM	4	3	3	3	3	3
*P*-value		0.82	0.82	0.84	0.65	0.64	0.95
2PD 2nd digit	WI	3	3	3	3	3	2
	AM	3.5	3	3	2.5	2	2
*P*-value		0.33	0.87	0.94	0.81	0.61	0.72
2PD 3rd digit	WI	4	3	3	3	3	3
	AM	3.5	3	3	3	3	3
*P*-value		0.98	0.67	0.70	0.88	0.68	0.92
2PD 4th digit	WI	4	3	3	3	3	3
	AM	3.5	3	3	3	3	3
*P*-value		0.86	0.79	0.60	0.72	0.49	0.49
SWM 1st digit	WI	3.61	3.61	3.61	3.61	2.83	2.83
	AM	3.61	3.61	3.61	3.61	3.22	2.83
*P*-value		0.53	0.64	0.72	0.91	0.60	0.37
SWM 2nd digit	WI	3.61	2.83	2.83	2.83	2.83	2.83
	AM	3.61	2.83	2.83	2.83	2.83	2.83
*P*-value		0.92	0.54	0.82	0.98	0.94	0.59
SWM 3rd digit	WI	3.61	3.61	3.61	2.83	2.83	2.83
	AM	3.61	3.61	3.61	2.83	2.83	2.83
*P*-value		0.52	0.32	0.37	0.40	0.53	0.16
SWM 4th digit	WI	3.61	3.61	3.61	3.61	3.61	2.83
	AM	3.61	3.22	3.22	2.83	2.83	2.83
*P*-value		0.75	0.49	0.60	0.65	0.58	0.60

Pre, preoperative; 2PD, two-point discrimination; WI, wrist immobilization group; AM, active motion group.

**Table 4:** Outcome measures of VAS and self-evaluation.

	**Pre median**	**2 weeks median**	**1 month median**	**2 months median**	**3 months median**	**6 months median**
**VAS**						
WI	1	1	1	0	0	0
AM	1	2	1	0.50	0	0
*P*-value	0.71	0.03^*^	0.22	0.76	1.00	0.62
**BCTQ-S**						
WI	2.35	2.55	2.05	1.85	1.55	1.30
AM	2.25	2.50	2.15	1.80	1.50	1.30
*P*-value	0.75	0.73	0.75	0.80	0.56	0.98
**BCTQ-F**						
WI	2.10	2.35	1.90	1.75	1.45	1.25
AM	1.85	2.35	1.80	1.55	1.35	1.20
*P*-value	0.91	0.66	0.86	0.38	0.88	0.86
**DASH**						
WI	28.80	32.95	24.25	18.80	15.45	13.75
AM	35.00	37.40	29.60	21.30	17.85	14.35
*P*-value	0.20	0.29	0.33	0.41	0.19	0.44

Pre, preoperative; VAS, visual analog score; BCTQ-S, Boston Carpal Tunnel Questionnaire of symptom severity scale; BCTQ-F, Boston Carpal Tunnel Questionnaire of functional status scale; DASH, Disabilities of the Arm, Shoulder, and Hand score; WI, wrist immobilization group; AM, active motion group. ^*^ denotes results with statistical significance.

**Table 5:** Outcome measures regarding the occurrence of pillar pain.

	**2 weeks**	**1 month**	**2 months**	**3 months**	**6 months**
**WI**					
*n* (%)	1 (6.7)	1 (6.7)	0	0	–
**AM**					
*n* (%)	6 (42.9)	5 (35.7)	3 (21.4)	2 (14.3)	–
*P*-value	0.035^*^	0.08	0.10	0.22	–

WI, wrist immobilization group; AM, active motion group; n, number; –, means no pillar pain occurred. ^*^ denotes results with statistical significance.

## Discussion

This study assessed postoperative outcome measures of wrist immobilization for 2 weeks following ECTR, compared to active mobilization with no restriction. Our results demonstrated that wrist immobilization significantly improved postoperative pain and strength rehabilitation at early follow-up, whereas there was no significant difference between the two groups regarding postoperative sensibility, flexibility, and self-evaluation. Interestingly, there was no significant difference between both groups in any outcome measure at the final follow-up.

ECTR has increasingly become popular worldwide (1–3). Though significant emphasis has been put on the strengths and limitations between ECTR and open carpal tunnel release (OCTR) (5–8), the postoperative protocol has been largely understudied. To minimize the complications, some surgeon traditionally immobilizes the wrist in question for 1–4 weeks after OCTR (25, 26). An increasing body of evidence suggests that wrist immobilization following OCTR is ineffective and adversely affects postoperative rehabilitation (10–13, 16, 17, 19). However, relevant study concerning wrist immobilization following ECTR is rare. The published literature does not recommend wrist immobilization or not after ECTR. Due to a lack of thorough understanding, some trials allowed initial mobilization while others recommended a plaster splint casting at early stages with variable duration of immobilization (from 48 h to 2 weeks) (14, 15, 18).

In this study, the outcome measures of sensibility improved sustainably in both groups, especially 1 month after ECTR, consistent with the literature. All patients benefited from ECTR and showed no obvious indication of incomplete release. Moreover, the sensibility of both groups at every time point was comparable, which suggested compressive nerve recovery after release was not disturbed by external splint casting.

Postoperative pain peaked at 2 weeks and was initially alleviated at 1 month after ECTR in both groups. At 2 weeks after ECTR, the wrist immobilization group exhibited better outcomes in VAS and pillar pain occurrence than the active motion group. Although pillar pain occurrence between the two groups differed by almost 30% at 1 month postoperatively, this difference did not reach statistical significance, which might be attributed to inadequate sample size. Subsequently, the two groups showed comparable outcomes regarding postoperative pain and achieved satisfactory results at the final follow-up. This difference could be related to stimulation by tiny subcutaneous hematoma and palmar cutaneous branches (27). The premature active motion can reportedly delay nerve recovery and swelling regression. In contrast, wrist immobilization for 2 weeks favored alleviation of the micro-circumstances and relief of nerve edema.

The change in postoperative strength showed a similar tendency to postoperative pain (13, 28). It took nearly 2 months for both groups to recover normal strength, which could provide a reference for the therapist to formulate accurate postoperative physiotherapy. As stated, the wrist immobilization group yielded greater strength than the active motion group at 2 weeks after ECTR. Various factors accounted for this difference although postoperative pain was the predominant cause. After pain alleviation and wound healing, wrist strength got improved 1 month after ECTR. At the final follow-up, the strength of both groups was stronger than the baseline, which was attributed to the recovery of the median nerve and the corresponding innervated muscles.

Postoperative flexibility and self-evaluation demonstrated no significant difference between these two groups in the whole follow-up. The DASH outcome measures at 2 weeks after ECTR was superior in the wrist immobilization group although this difference was not significant. This finding implied that pain and slight strength did not influence the daily routine.

No severe complications like bowstringing of the flexor tendons occurred, which showed the safety of ECTR. In total, two subjects in the wrist mobilization group complained of scar discomfort, which got worse during grip. Moreover, they both felt discomfortable with a scar on the palm rather than the forearm. Despite a lack of theoretical support, we considered that premature active motion, especially grip, would stimulate the cutaneous nerve in this region and cause discomfort.

The outcome measures of ECTR in this study are in accordance with the literature (29–32). This is the first study to compare the outcome of wrist immobilization and active motion after ECTR, which provides novel insights. Earlier studies have demonstrated little value of wrist immobilization after OCTR, and the outcomes even worsened with early immobilization. As already mentioned, splint casting would weaken the strength and cause stiffness of the wrist, which in turn delayed rehabilitation (13). However, we found that wrist immobilization is necessary for the early stage and contributes to symptom relief at 2 weeks postoperatively. This discrepancy may be attributed to the difference in the surgical technique. Due to the limited visualization, ECTR was considered with a higher risk of nerve damage compared with OCTR (5). Early immobilization may contribute to the recovery of the nerve. However, this damage is almost reversible and transient, and the symptoms will relieve with time pass in both groups. Further studies should be carried out to explain this discrepancy clearly.

Several limitations in this study should be given attention. First, the sample size was relatively small, which could easily cause bias and error. Moreover, this is a single-center study, and the findings only represent this single-center, which limits the generalization of our conclusions. Some outcome measures assessed are subjective, while objective outcome measures, such as EMG, were not analyzed. Hence, further multiple-center studies with large sample sizes are warranted for a more comprehensive investigation of outcome measures.

## Conclusion

Wrist immobilization for 2 weeks contributes to pain alleviation and improved grip and pinch strength during early follow-up. It brings no significant benefits in terms of improvement of sensibility, flexibility, and self-evaluation. At the final follow-up, wrist immobilization yielded no obvious significance compared with initial active motion. Therefore, wrist immobilization for 2 weeks is warranted after ECTR of idiopathic carpal tunnel syndrome.

## Data availability statement

The original contributions presented in the study are included in the article/supplementary material, further inquiries can be directed to the corresponding authors.

## Ethics statement

The studies involving human participants were reviewed and approved by Ethics Committee of WuXi No. 9 People's Hospital Affiliated to SooChow University. The patients/participants provided their written informed consent to participate in this study.

## Author contributions

GZ and YR designed this study. HJ, ZL, HY, QZ, and JM worked on data acquisition. FZ and HJ created tables and charts. FZ analyzed the data and drafted this manuscript. GZ revised and finished this manuscript. All authors contributed to the article and approved the submitted version.
